# Cephalometric Hard and Soft Tissue Norms in Lebanese Adults

**DOI:** 10.7759/cureus.85437

**Published:** 2025-06-05

**Authors:** Maria Saadeh, Ramzi Haddad, Maria Haydar, Fouad Ayoub

**Affiliations:** 1 Department of Orthodontics and Dentofacial Orthopedics, Lebanese University Faculty of Dental Medicine, Beirut, LBN; 2 Department of Forensic Odontostomatology and Human Identification, Lebanese University Faculty of Dental Medicine, Beirut, LBN; 3 Department of Dentofacial Medicine, American University of Beirut Medical Center, Beirut, LBN

**Keywords:** cephalometric measurements, hard tissue norms, lebanese adults, lebanese population, soft tissue norms

## Abstract

Background

Cephalometric norms are commonly used in orthodontic practice and research for guidance in diagnosis and treatment planning. They are related to race and ethnicity, thus making classical norms not applicable to all populations.

Objectives

The purpose of this study was to: present the cephalometric hard (HT) and soft tissue (ST) norms for adults with well-balanced faces and normal occlusion and evaluate the presence of sexual dimorphism within these measurements.

Material and methods

The sample consisted of pre-treatment cephalograms of 165 adult Lebanese subjects (59 males and 106 females, with a mean age of 23.6 ± 7.6 years) with balanced profiles and normal occlusion. Selected cephalometric hard and soft tissue measurements were automatically generated using Dolphin Imaging software. Statistical analyses included descriptive statistics for all the measurements and independent samples *t-*tests to evaluate gender differences.

Results

Adults with harmonious occlusion and profile exhibited orthognathic jaw relationship and a normodivergent pattern. The inclinations of maxillary and mandibular incisors were comparable to established Caucasian norms. ST facial angle indicated a straight profile in both genders (92.68±2.89 in males and 92.65±3.11 in females), with a nasolabial angle slightly more obtuse compared to Caucasian values (109.03±12.05 in males and 109.15±8.84 in females). Upper and lower lip relationship to E-plane indicated a straight subnasal profile, especially in males. As for gender differences, significantly larger skeletal and soft tissue linear measurements were observed in males (*p*≤0.05). The major differences resided in thicker and longer upper and lower lips (*p*≤0.001), more protrusive lips as assessed by lip protrusion and distance to H-line (*p*≤0.036), and smaller nasofrontal angle and Z-angles (*p*≤0.036).

Conclusions

Soft and hard tissue norms in Lebanese adults with pleasant profiles and normal occlusion are comparable to those of Caucasian populations except for jaws that are more retrognathic but smaller in dimensions and a more obtuse nasolabial angle. Gender dimorphism was found in linear skeletal and soft tissue measurements, in addition to larger nasofrontal and Z-angles in Lebanese males.

## Introduction

Orthodontic diagnosis and treatment planning are individualized procedures that result from a thorough assessment of a patient’s smile, facial and profile esthetics, and dental malocclusion. The orthodontic interest in the facial profile and its relation to the underlying skeletal structure has brought about decades of research into the “ideal” craniofacial features [1-8], and a cephalometric analysis has become standard practice in the assessment of patients for orthodontic treatment. With this objective in mind, different cephalometric analyses were introduced in the orthodontic field, namely, those by Downs, Steiner, Tweed, Ballard, Sassouni, Ricketts, Jacobson, and McNamara, from 1949 till the mid-eighties of the past century. Using these analyses, orthodontists would be able to compare the patient’s skeletal and dental relationships to those expected for the corresponding racial and ethnic background.. Furthermore, realizing that the overlying soft tissue does not accurately reflect the underlying skeletal structures, various soft tissue cephalometric analyses have been proposed as essential adjuncts to skeletal cephalometric analysis [9-13], mainly through the studies of Ricketts, Holdaway, Farkas, and McNamara.

Although early investigations by pioneer researchers were based on Caucasian and/or European population samples, later investigations into the cephalometric features of other populations have highlighted considerable variations between various racial and ethnic groups in addition to age- and gender-related differences [14-17], thus emphasizing the necessity of utilizing population-, gender- and age-specific norms.

Research on the cephalometric norms of the various populations in the Middle East and Arab region, although gaining momentum, remains in its infancy. Scarce assessments have been made in Jordanians [18], Yemenis [19], Syrians [20], Palestinians [21], Kuwaitis [22], Saudis [23], Emiratis [24,25], and Sudanese [26,27]. Some of these studies were conducted on limited samples [18,21-24] or focused on one gender or on a specific malocclusion [18-20]. In a study on hard and soft tissue measurement using lateral cephalometric radiographs of 30 Emirati adult males and 31 Emirati adult females, the results showed greater bimaxillary incisor proclination and protrusion compared to Caucasians [24]. Another study comparing Emirati men and women on soft tissue norms using lateral cephalograms of 176 adults with normal occlusion [25] showed a significant difference between the genders for most of the linear soft tissue measurements, with men displaying greater linear measurements and greater H-angle than women [25].

Investigations carried out on the Lebanese population are similarly scarce and have not sufficiently documented the vast array of craniofacial hard tissue norms that have been described by key investigators in their cephalometric analyses. While a few studies have assessed a limited number of skeletal norms [28-30], none have assessed the soft tissue profile in the Lebanese population. Ayoub et al. assessed hard tissue measurements based on lateral cephalograms of 63 individuals with normal occlusion of Lebanese origin and found that skeletal and linear measurements were significantly larger in males than in females [29]. More recently, and also assessing skeletal measurements in both genders in 117 Lebanese individuals with different malocclusions, Daraze et al. found that women had more convex faces and smaller skeletal structures than men [28].

The purpose of this study was to present the cephalometric hard and soft tissue norms for Lebanese adults with well-balanced faces and normal occlusion and evaluate the presence of sexual dimorphism within these measurements.

## Materials and methods

Study population

This is a cross-sectional study of the pre-treatment cephalograms of 165 adult Lebanese subjects (59 males, 106 females), with an age range of 16 to 45.91 years (mean age 23.6 ± 7.6 years). Radiographs were selected from the database of patients at the Faculty of Dental Medicine, Lebanese University, Beirut, Lebanon. The cephalograms were all taken using the same machine (Kodak 8000C, Carestream Health, Rochester, NY, USA) as part of standard pre-treatment orthodontic records collected between 2019 and 2024. Radiographs were assessed by the authors, and only those with the minimum double image at the level of the mandibular plane and the orbit and with visible and identifiable soft tissue and hard tissue landmarks were considered.

**Figure 1:**
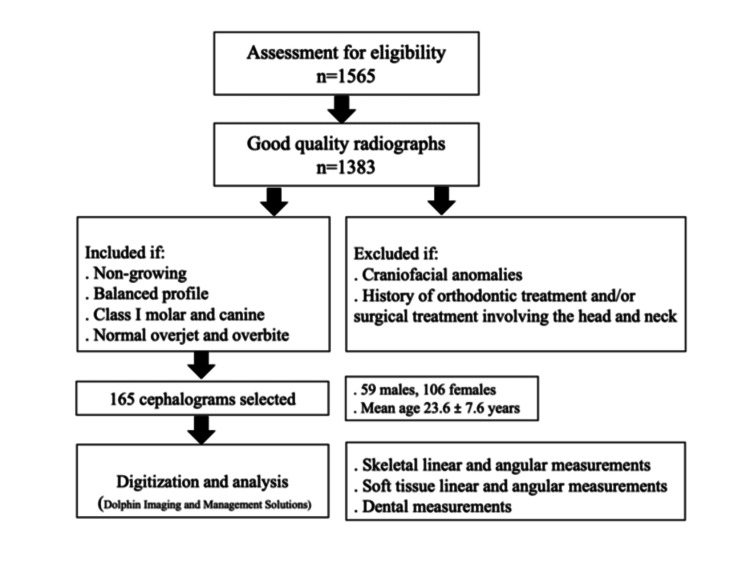
Flowchart of participant selection for the study

Inclusion criteria were non-growing subjects (age >16 years for females and >18 years for males), class I molar and canine dental relationships, and normal overjet (2±2 mm) and overbite (20-30% of the height of the mandibular incisors). Soft tissue profiles were classified as balanced and poorly balanced by three authors, and those cases in which total agreement on a balanced profile was achieved were selected for the study (Figure 1).

Subjects with craniofacial anomalies, history of orthodontic treatment, and/or surgical treatment involving the head and neck were excluded. The study was approved by the Institutional Review Board at the Lebanese University (CUEMB 35/AA).

Radiographic analysis

**Figure 2:**
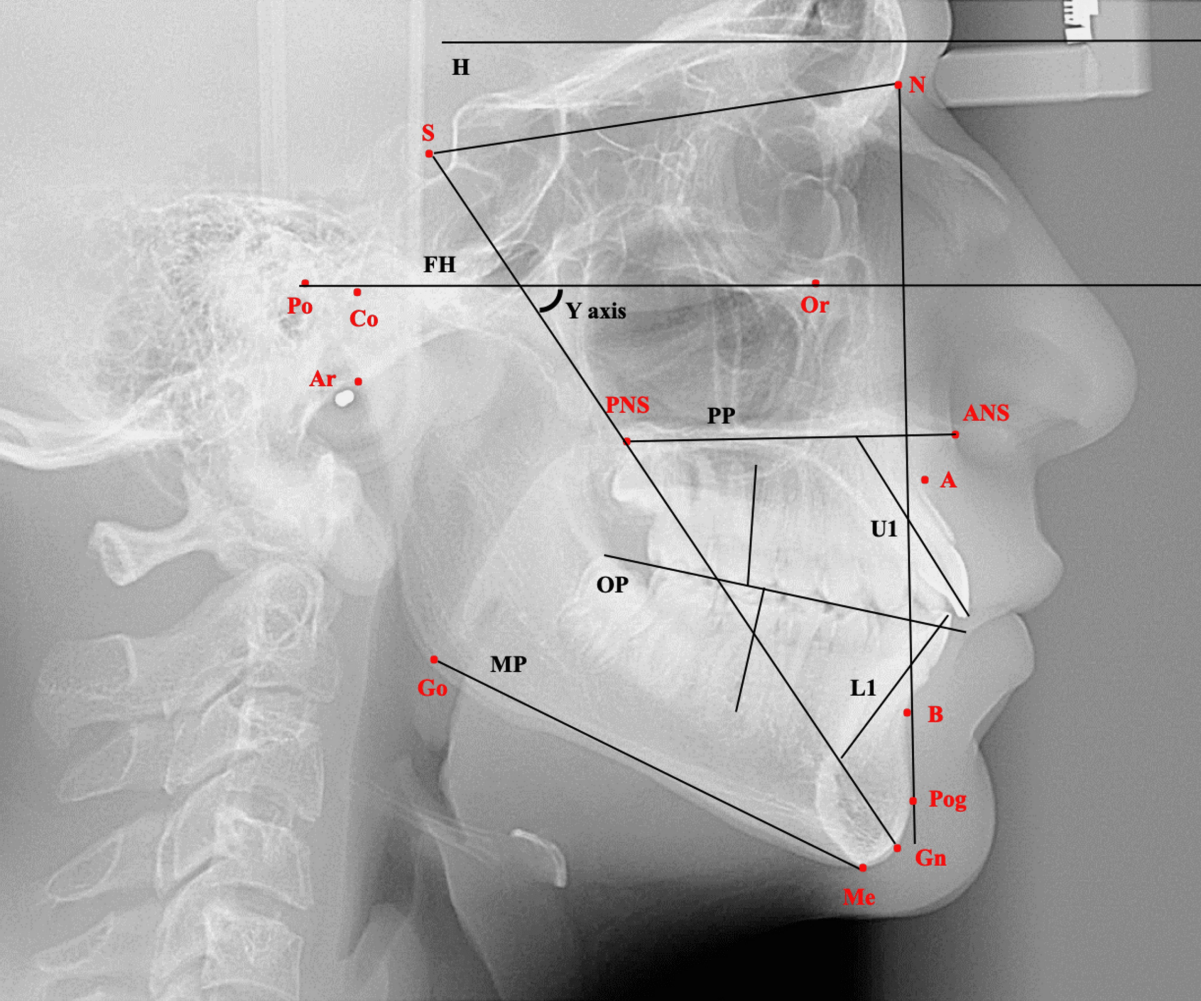
Cephalometric landmarks and planes. S, sella (center of the pituitary fossa); N, nasion (junction of the frontal and nasal bones); ANS, anterior nasal spine (tip of the bony anterior nasal spine at the inferior margin of the piriform aperture in the midsagittal plane); PNS, posterior nasal spine (most posterior point on the bony hard palate in the midsagittal plane); A, A point (deepest midline point on the curvature between the ANS and the dental alveolus); B, B point (deepest midline point on the bony curvature of the anterior mandible); Pog, pogonion (the most anterior point on the contour of the bony chin in the midsagittal plane); Me, menton (most inferior point on the chin in the lateral view); Go, gonion (most posterior inferior point on the outline of the angle of the mandible); Gn, gnathion (the most anterior inferior point of the bony chin); Po, porion (the most superior point located on the external auditory meatus); Or, orbitale (the lowest point on the infraorbital margin); H, true horizontal plane; PP, palatal plane (line connecting the ANS and PNS); MP, mandibular plane (line joining the Go and Me); FP, facial plane (line connecting the N and Pog); FH, Frankfurt horizontal (line joining the Po and Or); OP, occlusal plane (line extending through premolars and molars); Y axis, angle formed by the intersection of S-Gn with FH.

**Figure 3:**
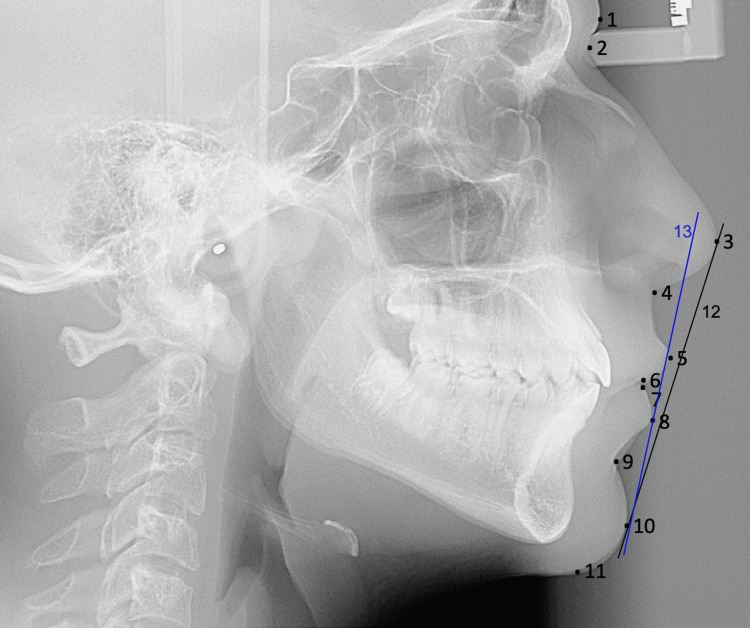
ST cephalometric landmarks and lines. 1. G’ (soft tissue glabella). 2. Na’(soft tissue nasion). 3. Pn (pronasale). 4. Sn (subnasale). 5. Ls (labrale superior). 6. StSup (stomium superior). 7. StInf (stomium inferior). 8. Li (labrale inferior). 9. B’ (soft tissue B point). 10. Pg’(soft tissue pogonion). 11. Me’ (soft tissue menton). 12. E line (line joining the Pn and Pg’). 13. H line (line connecting the Pg’ and Ls).

**Figure 4:**
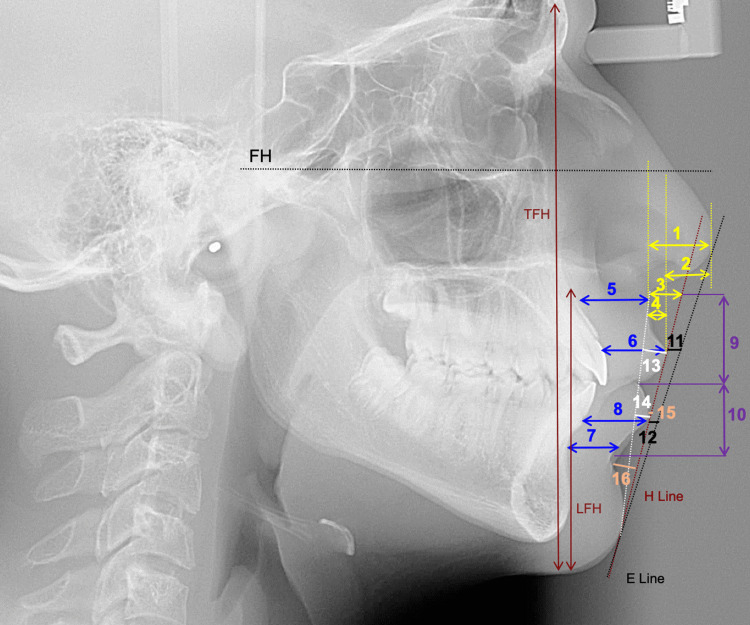
ST linear measurements. 1. Nasal projection (distance between a line tangent to Sn and a line tangent to Pn). 2. Nasal prominence (distance from a line perpendicular to the Frankfurt horizontal (FH) and tangent to the vermilion border of the upper lip to the tip of the nose). 3. Subnasale to H line (line tangent to the Pg’ and upper lip). 4. Superior sulcus depth (distance between the upper lip sulcus and a perpendicular line drawn from the vermilion to FH). 5. Upper lip thickness at A point. 6. Upper lip thickness at vermilion border. 7. Lower lip thickness at B point. 8. Lower lip thickness at the vermilion border. 9. Upper lip length (distance between the Sn and StSup). 10. Lower lip length (distance between StInf and B’). 11. Upper lip to the E line. 12. Lower lip to the E line. 13. Upper lip protrusion (distance between Sn-Pg’ and the most prominent point of the upper lip). 14. Lower lip protrusion (distance between Sn-Pg’ and the most prominent point of the lower lip). 15. Lower lip to the H line. 16. Inferior sulcus to the H line (distance at the point of maximum curvature on the lower lip and the H line). Lower face (%) is defined as the ratio between LFH (distance from Sn to soft tissue Me) and TFH (distance from soft tissue G to soft tissue Me). LFH, lower facial height; TFH, total facial height

**Figure 5:**
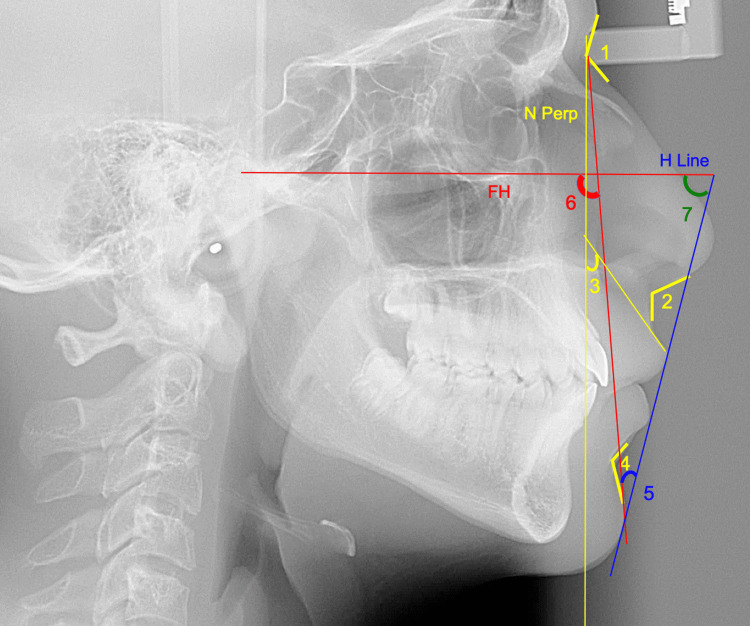
ST angular measurements. 1. Nasofrontal angle formed by the line tangent to the frontal lobe and a line tangent to the slope of the nose. 2. Nasolabial angle formed by the line tangent to the base of the nose and a line tangent to the upper lip. 3. Upper lip inclination formed by the line tangent to the upper lip and nasion perpendicular. 4. Mentolabial angle formed by the line tangent to the lower lip and the line tangent to the slope of the soft tissue chin. 5. H-angle formed by the line tangent to Pg’ and upper lip (H line) and the line between Na’ and Pg’. 6. ST facial angle formed by the line between Na’-Pg’ and Frankfurt horizontal (FH). 7. Z-angle formed by the line tangent to Pg’ and the most protrusive lip (Z line) and FH.

Lateral cephalometric radiographs taken in natural head position were imported into the Imaging program (Version 11.8, Dolphin Imaging and Management Solutions, La Jolla, CA, USA), where they were digitized by a single investigator (R.H.). Figure 2 represents the image view provided while digitizing using the Dolphin Imaging. Selected cephalometric hard and soft tissue measurements from various references were then automatically generated (Figures 2-5).

Descriptive statistics were generated for all cephalometric soft tissue (CHT) and cephalometric hard tissue (CST) measurements in the entire sample and in males and females separately. Shapiro’s Wilk’s test of normality showed that the data were normally distributed (p > 0.05). Independent t-tests were carried out to evaluate differences in CST and CHT between genders.

To assess intra-observer reliability, all measurements were repeated on 10 randomly selected radiographs at least 14 days after the initial assessment. The repeated measures were evaluated with the two-way mixed effects intra-class correlations for absolute agreement on single measures.

The power of the study was calculated using the G*Power 3.1.9.7 software to check if the sample of 165 adult Lebanese (59 males and 106 females) was adequate. With alpha set at 0.05 and an effect size of 0.5, the power was estimated at 0.86, which is satisfactory for presenting conclusive findings. Data were processed using the Statistical Package for Social Sciences (SPSS) version 23.0 (IBM Corp., Armonk, NY) and Stata/SE™ 11.1 (StataCorp LLC, College Station, TX). Statistical significance was set at 0.05.

## Results

Measurement reliability

Reliability of repeated measurements within and between operators was high, with correlation coefficients ranging from 0.976 to 0.996.

Hard tissue norms

Skeletal Norms

On average, the Lebanese sample with esthetically pleasing faces had maxillary and mandibular jaws that were both orthognathic compared to established Caucasian norms (SNA = 81.39 ± 4.15 and 81.28 ± 3.24 in males and females, respectively, and SNB = 79.39 ± 3.99 and 79.18 ± 3.31 in males and females, respectively), with an ANB angle of 2.00 ± 1.27 and 2.10 ± 1.14 in males and females, respectively, reflective of a skeletal class I relationship (Table 1). While mean AOBO was within 1 standard deviation of the norms in both males and females, the female average was reflective of a tendency towards concavity compared to Caucasians (-1.24 ± 2.39). Vertical divergence was also normal in both males and females as assessed by PP-MP (25.51 ± 5.39 and 24.50 ± 5.12, respectively), MP-SN (34.24 ± 6.21 and 33.85 ± 5.50, respectively), and FMA (25.16 ± 5.15 and 24.58 ± 4.74, respectively).

**Table 1:** Descriptive statistics for cephalometric hard tissue norms in the Lebanese population (n = 205) Measurements are reported in mean ± standard deviation (SD), and differences are reported in mean ± standard error of the mean (SE) *Statistically significant, p<0.05; **statistically significant, p<0.01. U1, maxillary incisor; L1, mandibular incisor; U6, maxillary first molar; L6, mandibular first molar

	Present Study			Males vs. Females		Established Norms	
	Males (n=99)	Females (n=106)	Difference	t	p	Males	Females
Skeletal							
SN-H (°)	9.60±4.12	10.52±3.50	-0.92±0.61	-1.506	0.134	9±2	9±2
SN-Ar (°)	124.43±5.55	125.30±5.47	-0.88±0.90	-0.976	0.331	124±5	124±5
SN (mm)	68.81±4.51	65.53±2.90	3.29±0.66	5.010	<0.001^**^	65.28±3	65.28±3
S-Ar (mm)	35.29±4.45	31.97±3.14	3.32±0.66	5.022	<0.001^**^	24.94±4	24.94±4
SNA (°)	81.39±4.15	81.28±3.24	0.11±0.63	0.170	0.866	82±3.5	82±3.5
SNB (°)	79.39±3.99	79.18±3.31	0.22±0.58	0.369	0.713	80.9±3.4	80.9±3.4
ANB (°)	2.00±1.27	2.10±1.14	-0.10±0.19	-0.534	0.594	1.6±1.5	1.6±1.5
AOBO (mm)	-1.23±2.55	-1.24±2.39	0.02±0.40	0.038	0.970	-1±1.9	0±1.77
NA-APo (°)	2.40±3.34	2.16±2.96	0.24±0.51	0.474	0.636	0±5.1	0±5.1
A-NPo (mm)	1.17±1.62	0.97±1.33	0.20±0.25	0.811	0.419	0.1±2	0.1±2
N-ANS (mm)	52.19±4.31	49.98±3.24	2.21±0.65	3.407	0.001^**^	50±2.5	50±2.5
ANS-Me (mm)	65.57±6.25	58.41±5.04	7.16±0.96	7.485	<0.001^**^	60±4.5	65±4.5
L/TFH (%)	57.46±2.38	55.80±2.32	1.66±0.38	4.315	<0.001^**^	55±1	55±1
PP-MP (°)	25.51±5.39	24.50±5.12	1.01±0.85	1.180	0.240	25±6	25±6
SN-PP (°)	8.72±3.96	9.35±3.69	-0.63±0.62	-1.015	0.312	7.3±3.5	7.3±3.5
MP-SN (°)	34.24±6.21	33.85±5.50	0.39±0.94	0.412	0.681	32.4±4.7	32.4±4.7
PP-H (°)	1.72±3.96	2.35±3.69	-0.63±0.62	-1.015	0.312	0.5±3	0.5±3
MP-H (°)	27.24±6.21	26.85±5.50	0.39±0.94	0.412	0.681	25±5	25±5
FMA (°)	25.16±5.15	24.58±4.74	0.58±0.80	0.726	0.469	23.9±4.5	23.9±4.5
Y-axis (°)	59.16±2.88	58.07±2.90	1.08±0.47	2.291	0.023^*^	60±3	60±3
FP-SN	80.23±3.96	80.23±3.43	-0.01±0.59	-0.009	0.993	80.5±4	80.5±4
Co-Gn (mm)	114.57±7.50	107.36±6.84	7.20±1.16	6.221	<0.001^**^	105.5±1.5	105.5±1.5
Co-Go (mm)	57.85±6.70	53.01±5.36	4.84±0.96	5.045	<0.001^**^	66±4	57±3
Go-Gn (mm)	79.04±6.26	76.07±5.77	2.97±0.97	3.050	0.003^**^	75.2±4.4	75.2±4.4
Go-Me (mm)	70.21±6.28	67.24±5.60	2.98±0.96	3.110	0.002^**^	71±5	71±5
ANS-PNS (mm)	54.33±5.06	50.95±4.07	3.38±0.73	4.645	<0.001^**^	51.6±4.3	51.6±4.3
Co-A (mm)	83.55±5.53	79.98±4.83	3.57±0.83	4.287	<0.001^**^	93.2±4	93.2±4
Co-Go-Me (°)	120.98±6.05	120.28±5.92	0.70±0.98	0.720	0.473	125±0.5	125±0.5
Dental							
U1-NA (mm)	4.35±1.92	4.16±2.01	0.19±0.32	0.588	0.558	4±2.7	4±2.7
U1-NA (°)	23.91±5.97	23.14±5.68	0.77±0.95	0.815	0.416	22.8±5.7	22.8±5.7
U1-SN (°)	105.29±7.10	104.42±6.42	0.88±1.09	0.804	0.423	102.8±5.5	102.8±5.5
U1-PP (°)	114.03±6.35	113.77±6.92	0.26±1.10	0.240	0.810	110±6	110±6
U6-PP (mm)	23.96±3.28	20.83±2.68	3.13±0.48	6.585	<0.001^**^	23±2	23±2
L1-APo (°)	23.91±4.74	24.89±5.70	-0.98±0.88	-1.110	0.268	22±4	22±4
L1-APo (mm)	2.53±2.10	2.02±2.14	0.51±0.35	1.479	0.141	2.7±1.7	2.7±1.7
L1-NB (°)	24.31±5.86	24.96±6.14	-0.65±0.99	-0.653	0.515	25.3±6	25.3±6
L1-NB (mm)	4.41±1.98	3.99±2.06	0.42±0.33	1.261	0.209	4±1.8	4±1.8
L1-MP (°)	90.68±6.98	91.93±6.89	-1.25±1.13	-1.100	0.273	95±7	95±7
L6-MP (mm)	29.36±3.03	26.20±2.70	3.17±0.46	6.862	<0.001^**^	31±2	31±2
U1-L1 (°)	129.77±9.72	129.81±10.26	-0.04±1.65	-0.026	0.979	130±6	130±6
Overbite (mm)	1.53±1.61	2.00±1.43	-0.46±0.24	-1.886	0.061	2.5±2	2.5±2
Overjet (mm)	2.90±1.27	3.14±0.98	-0.24±0.18	-1.366	0.174	2.5±2.5	2.5±2.5

Dental Norms

Maxillary incisor inclination and protrusion were comparable to the established Caucasian norms, although mean inclination to the palatal plane was slightly larger and indicative of slight proclination relative to Caucasians (114.03 ± 6.35 and 113.77 ± 6.92 in males and females, respectively). Lower incisors were on average well-inclined both relative to NB (24.31 ± 5.86 and 24.96 ± 6.14 in males and females, respectively) and to the mandibular plane (90.68 ± 6.98 and 91.93 ± 6.89 in males and females, respectively). The mean interincisal angle was 129.77 ± 9.72 and 129.81 ± 10.26 in males and females, respectively.

Soft tissue norms

Mean linear soft tissue cephalometric measurements ranged from 0.47 ± 1.28 mm for the lower lip to H-line to 19.95 ± 2.75 mm for the upper lip length, whereas mean angular measurement ranged from 5.9 ± 7.35 for upper lip inclination to 133.51 ± 10.26 for the nasofrontal angle. All mean measurements were within 1 standard deviation of the established cephalometric norms described for the cephalometric assessment of Caucasian populations (Table 2).

**Table 2:** Descriptive statistics for cephalometric soft tissue norms in the Lebanese population (n = 205) All linear measurements were recorded in (mm), and all angular measures were recorded in degrees. Measurements are reported in mean ± standard deviation (SD), and differences are reported in mean ± standard error of the mean (SE). *Statistically significant, p<0.05; ** statistically significant, p<0.01. UL, upper lip; LL, lower lip; VB, vermilion border

	Present Study			Males vs. Females		Established Norms	
	Males (n=99)	Females (n=106)	Difference	t	p	Males	Females
Linear measurements							
Nasal projection	18.87±2.06	18.26±1.87	0.61±0.32	1.937	0.054	18±2	18±2
Nasal prominence	13.44±2.35	13.06±2.36	0.38±0.39	0.991	0.323	15.3±3	15.3±3
Subnasale to the H-line	4.19±2.01	3.51±1.76	0.68±0.30	2.237	0.027^*^	4.2±2	4.2±2
Superior sulcus depth	2.80±1.22	2.70±0.98	0.10±0.19	0.545	0.587	3±1	3±1
UL thickness at A point	16.70±1.64	14.35±1.84	2.35±0.29	8.108	<0.001^**^	17±3	17±3
UL thickness at VB	13.37±1.99	11.20±1.70	2.16±0.30	7.307	<0.001^**^	13.9±3	13.9±3
LL thickness at B point	11.87±1.67	10.67±1.25	1.20±0.25	4.792	<0.001^**^	1	1
LL thickness at VB	14.57±1.50	12.68±1.55	1.90±0.25	7.549	<0.001^**^	15.1±1.2	13.6±1.4
UL length	21.65±2.78	19.01±2.25	2.64±0.40	6.591	<0.001^**^	22±1.8	20±1.6
LL length	20.10±2.64	17.69±2.32	2.42±0.40	6.066	<0.001^**^	20±1.5	19±1.7
UL to E-plane	-4.87±1.81	-5.12±1.64	0.25±0.28	0.911	0.364	-4±2	-4±2
LL to E-plane	-2.29±1.84	-2.79±1.74	0.50±0.29	1.720	0.087	-2±2	-2±2
UL protrusion	3.11±1.52	2.63±1.29	0.48±0.23	2.115	0.036^*^	3±1	3±1
LL protrusion	2.70±1.59	1.90±1.64	0.80±0.27	3.030	0.003^**^	1.5±1.5	1.5±1.5
LL to H-line	0.76±1.21	0.30±1.29	0.46±0.21	2.224	0.028^*^	0.7±2	0.7±2
Inferior sulcus to H-line	4.91±1.16	4.46±1.24	0.45±0.20	2.260	0.025^*^	4±2	4±2
Angular measurements							
Nasofrontal angle	130.89±11.59	134.96±9.19	-4.07±1.77	-2.306	0.023^*^	130.3±7.4	134.3±7
Nasolabial angle	109.03±12.05	109.15±8.84	-0.12±1.80	-0.068	0.946	102±8	102±8
UL inclination	5.67±7.91	6.03±7.06	-0.36±1.21	-0.299	0.765	8±8	14±8
Mentolabial angle	133.64±10.93	133.30±11.69	0.34±1.87	0.181	0.857	127±11.6	133±13
H-angle	13.45±3.18	12.71±2.83	0.75±0.48	1.546	0.124	10±4	10±4
ST facial angle	92.68±2.89	92.65±3.11	0.03±0.50	0.051	0.959	91.6±7	91.6±7
Z-angle	77.47±5.68	79.37±5.36	-1.89±0.90	-2.113	0.036^*^	80±9	80±9
Other measurements							
Lower face (%)	54.06±2.75	52.06±2.25	2.00±0.42	4.743	<0.001^**^	55±1.5	54±1.5

Both Lebanese male and female faces displayed a mean nasofrontal angle that is compatible with Caucasian norms (130.89 ± 11.59 and 134. 96 ± 9.19, respectively) and a nasolabial angle that is slightly more obtuse when compared to the Caucasian means, nonetheless still within 1 standard deviation (109.03 ± 12.05 and 109.15 ± 8.84, respectively). While mean nasal projection in our sample was very similar to that described in Caucasians (18.87 ± 2.06 and 18.26 ± 1.87, respectively), mean nasal prominence was 1.86-2.24 mm smaller than the Caucasian norm (13.44 ± 2.35 and 13.06 ± 2.36, respectively).

Thickness and length of the upper and lower lips were compatible with previously established Caucasian norms in both genders. In females, however, mean values for upper lip thickness were toward the lower end of 1 standard deviation of the norms (14.35 ± 1.84 and 11.20 ± 1.70 for thickness at A-point and at the vermilion border, respectively). Upper lip inclination in our sample was within 1 standard deviation of Caucasian norms, but mean values were in comparison smaller in both males and females (5.67 ± 7.91 and 6.03 ± 7.06, respectively), indicating a flatter, more retroclined upper lip. The mental sulcus, as described by the inferior sulcus to H-line (4.91 ± 1.61 and 4.46 ± 1.24, respectively) and by the mentolabial angle (133.64 ± 10.93 and 133.30 ± 11.69, respectively), was very similar to the described Caucasian norms in both males and females.

Facial convexity, as described by the ST facial angle, indicated a straight profile in Lebanese males and females (92.68 ± 2.89 and 92.65 ±3.11) as compared to Caucasian norms. The H-angle, while within 1 standard deviation of the norms, was slightly larger in both genders (13.45 ± 3.18 and 12.71 ± 2.83 in males and females, respectively), indicating a slightly more convex profile. Upper and lower lip relationship to the E-plane indicated a straight sub-nasal profile, especially in males (-4.87 ± 1.81 and -2.29 ± 1.84 for upper and lower lips, respectively).

Gender differences

As expected, most linear cephalometric skeletal measurements exhibited significant gender dimorphism, as illustrated by significantly larger cranial base, effective maxillary length, mandibular body and effective length and upper and lower facial heights in males compared to females (p < 0.05; Table 1). Mean difference ranged from 2.21 mm for N-ANS to 7.20 mm for Co-Gn. Among the linear skeletal measurements, only AOBO and A-NPo were similar between males and females (p ≥ 0.636). The only angular skeletal measurement that displayed gender differences was Down’s Y-axis, which was on average 1.08 degrees larger in males (59.16 ± 2.88 and 58.07 ± 2.90 in males and females, respectively; p = 0.023).

With respect to dental measurements, males and females had similarly inclined and positioned maxillary and mandibular incisors in the sagittal plane (p ≥ 0.141). The only dental variables that differed between genders were the vertical distance between the molars (maxillary and mandibular) and the palatal and mandibular planes (U6-PP = 23.96 ± 3.28 and 20.83 ± 2.68 in males and females, respectively, p < 0.001; L6-MP = 29.36 ± 3.03 and 26.20 ± 2.70 in males and females, respectively, p < 0.001).

The majority of the assessed linear soft tissue norms also displayed statistically significant gender dimorphism (p < 0.05; Table 2), the trend being for larger measurements in males by a difference that ranged from 0.45 mm for inferior sulcus to H-line (p = 0.025) to 2.64 mm for upper lip length (p < 0.001). Males had thicker and longer upper and lower lips (p < 0.001), and more protrusive upper and lower lips as assessed by lip protrusion and distance to H-line (p ≤ 0.036). Notably, nasal projection and prominence were similar in both genders (p ≥ 0.054) and so was superior sulcus depth (p = 0.545). Additionally, the relationship between both upper and lower lips to the E-line was also similar between males and females (p ≥ 0.087).

Out of the seven assessed angular measurements, only the nasofrontal and Z-angles showed statistically significant differences between males and females, with males presenting with smaller nasofrontal and Z-angles than females (p ≤ 0.036). The largest difference was noted for the nasofrontal angle, which was smaller by 4.07 degrees in males (p = 0.023).

## Discussion

Orthodontists have been studying the face in profile since Edward Angle’s first description of malocclusion more than a century ago, the result of which is the availability of a vast number of hard [1-7] and soft [9-13] tissue cephalometric analyses relying on a multitude of different measurements. While the majority of hard and soft tissue norms were initially described on Caucasian populations, both clinical practice and research have illustrated the inappropriateness of applying these norms to other populations [14,16,31,32]. Research efforts have increasingly been placed into assessing population-specific cephalometric norms in various ethnicities and countries, including several Arab and regional populations [15,24,33]. Previous assessments of the Lebanese population, however, are limited due to an incomplete assessment of only a few cephalometric variables and/or small sample sizes [28-30]. To our knowledge, this research represents the first comprehensive assessment of a variety of hard tissue cephalometric measurements and the absolute first assessment of soft tissue cephalometric norms in a large sample of the Lebanese population. The attempt to include various measurements from the most commonly utilized cephalometric analyses allowed the authors to compare Lebanese norms to those described in several other populations.

When compared to previous research, the sagittal positions of the maxilla and mandible, as represented by SNA and SNB in our sample, support the previous research by Daraze et al. on a sample of 117 adult Lebanese subjects that illustrates an orthognathic position of both jaws (Table 3) [28]. Earlier research on a smaller sample of 63 subjects, on the other hand, suggested smaller SNA and SNB angles reflective of retrognathic jaws, and a borderline skeletal class I relationship, as illustrated by mean ANB values of 4.03 ± 2.61 in males and 3.55 ± 2.83 in females. Linear jaw measurements (Co-Go and Co-A) in our sample, however, were more consistent with the norms presented by Ayoub et al. [29] and smaller than those described by Daraze et al. [28]. Our data also support the previously reported finding of normal vertical divergence in the Lebanese population as assessed by the PP-MP angle [29]. Mean values of maxillary incisor proclination were slightly larger than those previously described by Ayoub et al., who found the maxillary incisors to be at 111.87 ± 6.57 degrees to the palatal plane in males and at 111.48 ± 8.69 degrees in females [29]. Our findings also contradict the finding of slightly more proclined mandibular incisors relative to the mandibular plane, reported at 94.13 ± 16.84 in males and 96.42 ± 6.58 in females [29].

**Table 3:** Mean cephalometric hard tissue measurements in Lebanese males and females compared to other studies on the Lebanese population M, male; F, female; U1, maxillary incisor; L1, mandibular incisor

Authors	Current		Ayoub et al. [26,27]		Daraze et al. [25]	
Year of publication	2024		2008; 2009		2017	
	M	F	M	F	M	F
Skeletal						
SN (mm)	68.81	65.53	74.0	72.88	N/A	N/A
SNA (°)	81.39	81.28	79.5	76.71	81	80.9
SNB (°)	79.39	79.18	75.44	72.74	79.7	77.8
ANB (°)	2.00	2.10	4.03	3.55	1.2	3
N-ANS (mm)	52.19	49.98	53.93	53.3	N/A	N/A
ANS-Me (mm)	65.57	58.41	71.10	69.25	N/A	N/A
PP-MP (°)	25.51	24.50	23.87	25.06	N/A	N/A
Co-Gn (mm)	114.57	107.36	118.7	114.74	133.4	121.7
CoGo (mm)	57.85	53.01	N/A	N/A	N/A	N/A
Go-Me (mm)	70.21	67.24	75.68	72.80	N/A	N/A
Co-A (mm)	83.55	79.98	N/A	N/A	99.7	93.3
Co-Go-Me (º)	120.98	120.28	117.18	115.81	N/A	N/A
Dental						
U1-PP (º)	114.03	113.77	111.87	111.48	N/A	N/A
IMPA (º)	90.68	91.93	94.13	96.42	N/A	N/A
U1-L1 (º)	129.77	129.81	129.20	127.10	N/A	N/A
Overjet (mm)	2.90	3.14	N/A	N/A	4.4	4.2

The comparison of SNA and SNB angles to norms reported by similar studies on populations in the Middle East suggest that the Lebanese population is similar to the Turkish and Yemeni populations [15,19] and that the Kuwaiti, Saudi Arabian, and Sudanese populations possess slightly more prognathic maxillary and mandibular jaws [15,27,33] (Table 4). While Rabah et al. reported on a similar slight protrusion of the jaws in the Emirati population [34], the norms reported by Al Zain and Ferguson [24] are more consistent with the orthognathic jaws noted in the Lebanese, Yemeni, and Turkish populations. On the other hand, the linear dimensions of the jaws, as assessed by Co-Gn and Co-A, were smaller in our Lebanese sample than in the Turkish sample and more consistent with the dimensions reported on the Saudi Arabian and Sudanese populations [15,27]. Compared to our sample, the Yemeni population had a more hypodivergent vertical relationship of the jaws, as assessed by PP-MP [19], and so did the Kuwaiti population, as assessed by FMA and MP/SN [33].

**Table 4:** Mean cephalometric hard and soft tissue measurements in Lebanese males and females compared to other Arab and Middle Eastern populations M, male; F, female; UL, upper lip; LL, lower lip; U1, maxillary incisor; L1, mandibular incisor

Authors	Current		Younso et al. [27]		Abutayyem et al. [25]		Rabah et al. [31]		Al Zain and Ferguson [23]		Al-Qaisi et al. [17]		Daer and Abuaffan [18]		Al Awwad et al. [30]		Uysal et al. [15]		Uysal et al. [15]	
Population	Lebanese		Sudanese		Emirati		Emirati		Emirati		Qatari		Yemeni		Kuwaiti		Turkish		Saudi Arabian	
Year of publication	2024		2021		2021		2017		2012		2020		2016		2014		2011		2011	
	M	F	M	F	M	F	M	F	M	F	M	F	M	F	M	F	M	F	M	F
Skeletal																				
SNA(°)	81.39	81.28	83.27	82.2	N/A	N/A	84.35	83.03	82.5	80.8	N/A	82.68	81	80.8	83.3	83.38	80.5	80.2	83	82.5
SNB (°)	79.39	79.18	81.02	79.07	N/A	N/A	79.81	79.44	79.6	77.6	N/A	79.82	78	77.5	81.21	80.53	78.4	77.3	80.8	78.5
ANB (°)	2	2.1	2.43	3.16	N/A	N/A	4.53	3.58	3	3.3	N/A	2.96	2.6	3.3	2.13	2.86	2.1	2.9	2.5	4.1
AOBO (mm)	-1.23	-1.24	−1.22	−1.38	N/A	N/A	N/A	N/A	N/A	N/A	N/A	N/A	N/A	N/A	-0.93	-1.56	N/A	N/A	N/A	N/A
NA-APo (°)	2.4	2.16	N/A	N/A	N/A	N/A	N/A	N/A	N/A	N/A	N/A	N/A	N/A	N/A	1.83	4.67	N/A	N/A	N/A	N/A
A-NPo (mm)	1.17	0.97	N/A	N/A	N/A	N/A	N/A	N/A	N/A	N/A	N/A	N/A	N/A	N/A	1.47	2.69	N/A	N/A	N/A	N/A
N-ANS (mm)	52.19	49.98	N/A	N/A	N/A	N/A	N/A	N/A	N/A	N/A	N/A	N/A	N/A	N/A	N/A	N/A	58.1	55.3	52.8	50
SN-NPog (°)	80.23	80.23	N/A	N/A	N/A	N/A	N/A	N/A	N/A	N/A	­N/A	81.33	79	78.3	N/A	N/A	N/A	N/A	N/A	N/A
Y-axis (°)	59.16	58.07	N/A	N/A	N/A	N/A	N/A	N/A	N/A	N/A	N/A	N/A	N/A	N/A	56.71	57.49	N/A	N/A	N/A	N/A
ANS-Me (mm)	65.57	58.41	67.73	64.31	N/A	N/A	N/A	N/A	N/A	N/A	N/A	N/A	N/A	N/A	73.18	71.19	74.6	68.4	68.5	63.5
Co-Gn (mm)	114.6	107.4	115.06	107.35	N/A	N/A	N/A	N/A	N/A	N/A	N/A	N/A	N/A	N/A	N/A	N/A	125	116	119	108
Go-Gn (mm)	79.04	76.07	­­N/A	­N/A	N/A	N/A	N/A	N/A	N/A	N/A	N/A	N/A	N/A	N/A	126.2	127.2	N/A	N/A	N/A	N/A
Co-A (mm)	83.55	79.98	86	81.27	N/A	N/A	N/A	N/A	N/A	N/A	N/A	N/A	N/A	N/A	93.85	96.24	90.8	86.9	86.9	82.7
PP-MP (°)	25.51	24.5	22.22	26.07	N/A	N/A	N/A	N/A	24.8	25.2	N/A	N/A	20	22.4	N/A	N/A	N/A	N/A	N/A	N/A
SN-PP (°)	8.72	9.35	N/A	N/A	N/A	N/A	N/A	N/A	8.4	10.7	N/A	N/A	8.9	10.7	N/A	N/A	N/A	N/A	N/A	N/A
MP-SN (°)	34.24	33.85	29.57	34.65	N/A	N/A	31.39	31.91	33.3	35.9	N/A	N/A	29	33.1	25.53	28.81	31	32	30.5	34.5
FMA (°)	25.16	24.58	N/A	N/A	N/A	N/A	N/A	N/A	N/A	N/A	N/A	N/A	N/A	N/A	20.6	22.84	N/A	N/A	N/A	N/A
Dental																				
U1-NA (mm)	4.35	4.16	N/A	N/A	N/A	N/A	0.05	0.05	6	6.2	­N/A	5.23	4.7	4.5	5.16	5.96	3.9	3.9	6	3.6
U1-NA (°)	23.91	23.14	N/A	N/A	N/A	N/A	25.28	25.27	N/A	N/A	N/A	28.02	21	21.8	21.89	21.01	21.2	20.4	27.2	21.9
U1-SN (°)	105.3	104.4	109.14	106.13	N/A	N/A	N/A	N/A	N/A	N/A	N/A	N/A	N/A	­N/A	105.2	104.4	102	101	110	105
U1-PP (°)	114	113.8	116.51	114.84	N/A	N/A	N/A	N/A	116	116	N/A	N/A	N/A	­N/A	N/A	N/A	N/A	­N/A	N/A	­N/A
L1-APo (mm)	2.53	2.02	5.43	6.25	N/A	N/A	N/A	N/A	5.8	5.3	N/A	N/A	N/A	­N/A	2.91	4.85	1.5	2.3	4.4	3
L1-NB (°)	24.31	24.96	N/A	N/A	N/A	N/A	26.48	26	N/A	N/A	N/A	28.91	27	29.2	26.53	31.81	22.4	27.3	28.6	30.7
L1-NB (mm)	4.41	3.99	N/A	N/A	N/A	N/A	0.06	0.06	6.7	6.6	N/A	5.65	6	6.5	5.53	7.2	4.4	5	6.6	6
L1-MP (°)	90.68	91.93	101.12	101.65	N/A	N/A	N/A	N/A	101	99.8	N/A	N/A	N/A	N/A	96.49	99.33	89.8	95.2	95.9	96.4
U1-L1 (°)	129.8	129.8	119.94	117.47	N/A	N/A	122.6	122.6	118	119	N/A	122.28	127	126	129.5	124.3	134	129	120	121
Overbite (mm)	1.53	2	N/A	N/A	N/A	N/A	N/A	N/A	0.7	0.8	N/A	N/A	N/A	N/A	N/A	N/A	N/A	N/A	N/A	N/A
Overjet (mm)	2.9	3.14	N/A	N/A	N/A	N/A	N/A	N/A	3.3	4.3	N/A	N/A	N/A	N/A	N/A	N/A	N/A	N/A	N/A	N/A
Soft tissue																				
Nasolabial angle (°)	109	109.2	90.92	92.14	117.62	120.13	N/A	N/A	122	116	­N/A	99.33	N/A	N/A	109.4	111.3	N/A	N/A	N/A	N/A
UL to E-line (mm)	-4.87	-5.12	−0.39	−0.13	-5.88	-1.8	N/A	N/A	N/A	N/A	­N/A	3.32	N/A	N/A	-5.66	-5.4	-6.1	-5.8	-3.8	-4.1
LL to E-line (mm)	-2.29	-2.79	2.37	2.62	-2.23	-1.32	N/A	N/A	N/A	N/A	­N/A	1.19	N/A	N/A	-3.11	-0.99	-3.4	-2.6	-0.3	-1.7
UL protrusion (mm)	3.11	2.63	N/A	N/A	3.47	12.01	N/A	N/A	2.4	2.8	N/A	N/A	N/A	N/A	N/A	N/A	N/A	N/A	N/A	N/A
LL protrusion (mm)	2.7	1.9	N/A	N/A	3.72	11.75	N/A	N/A	4	2.64	N/A	N/A	N/A	N/A	N/A	N/A	N/A	N/A	N/A	N/A
H-angle (°)	13.45	12.71	N/A	N/A	14.56	12.99	N/A	N/A	N/A	N/A	N/A	N/A	N/A	N/A	N/A	N/A	12.7	12.7	16.3	15.4
Lower face (%)	54.06	52.06	­N/A	­N/A	­N/A	­N/A	­N/A	­N/A	49.9	50.8	­N/A	­N/A	­N/A	­N/A	62.89	55.56	N/A	N/A	N/A	N/A

The assessment of dental protrusion in our Lebanese sample confirms similarity to the Turkish population, where maxillary incisors to NA were found to be at 3.9 mm in both genders and mandibular incisor protrusion to A-Pog was found at 1.5 and 2.3 mm in males and females, respectively. The Emirati population assessed by Rabah et al. [34] had significantly more retruded maxillary incisors, measured at 0.05 mm to NA in both genders. The Kuwaiti sample assessed by Al-Awwad [33], in addition to the Emirati sample assessed by Al Zain and Ferguson [24], had considerably more protruded incisors measuring between 5 and 6 mm to NA. It is interesting to note that despite more protruded upper incisors relative to NA, the Emiratis presented larger nasolabial angles reported at 121.6 ± for males and 116.4 ± for females [24]. This apparent contradiction, however, may be the result of differences in lip thickness or nasal tip morphology. Also noteworthy is the general finding of a more retruded upper lip relative to E-line compared to the described norm of -4 mm [35] in the majority of the assessed populations in the region, as was the finding in our sample of Lebanese subjects, which is possibly reflective of a more prominent nose and chin in the region’s populations.

A brief comparison of the norms reported by a few Caucasian studies suggests that Lebanese adults have relatively more retrognathic jaws but a similar ANB angle, jaws that are smaller in dimension, incisors that are slightly more retroclined, and a fairly similar nasolabial angle [31,32]. Compared to Asian and South Asian populations, Lebanese adults have more retrognathic jaws that are similarly smaller in dimensions [31,32,36] (Table 5). The sagittal relationship between the jaws, however, was more orthognathic in the Lebanese sample compared to mean ANB values ranging from 2.8 ± 2 degrees in Japanese males to 3.9 ± 1.8 degrees in Chinese females. Reported Chinese and Filipino averages for maxillary incisor inclination to SN were considerably larger, indicative of more proclined incisors [31,36], and mandibular incisor protrusion was similarly greater in the Bangladeshi, Japanese, and Chinese populations than in the Lebanese [14,31]. Across all the assessed Asian populations, the nasolabial angle was more acute than in our Lebanese sample, especially in the Japanese and Korean samples [16,37]. This finding, along with smaller norms for upper and lower lip distances to E-plane and larger values for upper and lower lip protrusion, confirms the presence of significantly more protruded upper and lower lips in the Asian than in the Lebanese population.

**Table 5:** Mean hard and soft tissue cephalometric measurements in Lebanese males and females (n=205) compared to other studies on Asian and South Asian populations M, male; F, female; UL, upper lip; LL, lower lip; VB, vermilion border

Authors	Current		Ahsan et al. [14]		Gu et al. [28]		Loi et al. [29]		Miyajima et al. [16]		Hwang et al. [34]		Moldez et al. [33]	
Population	Lebanese		Bangladeshi		Chinese		Japanese		Japanese		Korean		Filipino	
Year of publication	2024		2013		2011		2007		1996		2002		2006	
	M	F	M	F	M	F	M	F	M	F	M	F	M	F
SN (mm)	68.81	65.53	N/A	N/A	N/A	N/A	N/A	N/A	N/A	N/A	N/A	N/A	74.9	69.7
SNA (°)	81.39	81.28	84.5	83.8	85.1	83.6	N/A	N/A	82.2	82.1	82.1	80.2	84.4	82.7
SNB (°)	79.39	79.18	83	81.7	81.6	79.7	N/A	N/A	79.4	78.8	79.5	77.9	81.2	78.7
ANB (°)	2	2.1	1.6	2.1	3.5	3.9	­N/A	­N/A	2.8	3.3	2.6	2.4	3.3	4
AOBO (mm)	-1.23	-1.24	N/A	N/A	-0.6	-1.1	N/A	N/A	­N/A	­N/A	­N/A	­N/A	­N/A	­N/A
NA-APo (°)	2.4	2.16	1.8	3	N/A	N/A	N/A	N/A	­N/A	­N/A	­N/A	­N/A	­N/A	­N/A
N-ANS (mm)	52.19	49.98	N/A	N/A	­N/A	­N/A	­N/A	­N/A	­N/A	­N/A	­N/A	­N/A	59.3	56.3
ANS-Me (mm)	65.57	58.41	N/A	N/A	74.9	69.4	74.8	71	­N/A	­N/A	­N/A	­N/A	75.5	69.4
SN-PP (°)	8.72	9.35	N/A	N/A	­N/A	­N/A	­N/A	­N/A	­N/A	­N/A	­N/A	­N/A	8.3	10.1
MP-SN (°)	34.24	33.85	N/A	N/A	­N/A	­N/A	­N/A	­N/A	­N/A	­N/A	­N/A	­N/A	31.8	36.1
FMA (°)	25.16	24.58	18.4	20.1	28.2	30.4	25.1	26.5	­N/A	­N/A	­N/A	­N/A	­N/A	­N/A
Y-axis (°)	59.16	58.07	58	57.8	­N/A	­N/A	­N/A	­N/A	­N/A	­N/A	­N/A	­N/A	60.6	61.3
Co-Gn (mm)	114.57	107.36	112.4	105.2	125.4	119.4	130.4	121.5	­N/A	­N/A	­N/A	­N/A	130.2	119.2
CoGo (mm)	57.85	53.01	N/A	N/A	­N/A	­N/A	­N/A	­N/A	­N/A	­N/A	­N/A	­N/A	67.3	58.6
Co-A (mm)	83.55	79.98	83.4	78.7	92	87.5	96.9	91.5	­N/A	­N/A	­N/A	­N/A	­N/A	­N/A
U1-SN (°)	105.29	104.42	­N/A	­N/A	114.6	115	­N/A	­N/A	­N/A	­N/A	­N/A	­N/A	111.4	108.2
U1-PP (°)	114.03	113.77	N/A	N/A	­N/A	­N/A	­N/A	­N/A	­N/A	­N/A	­N/A	­N/A	­N/A	­N/A
U6-PP (mm)	23.96	20.83	22.5	20.8	­N/A	­N/A	26.6	25.3	­N/A	­N/A	­N/A	­N/A	26.6	23.7
L1-APo (mm)	2.53	2.02	3.8	3.5	4.1	4.1	4.2	4.9	­N/A	­N/A	­N/A	­N/A	­N/A	­N/A
L1-MP (°)	90.68	91.93	101.3	99.7	94.4	94.5	97.8	99.5	­N/A	­N/A	­N/A	­N/A	99.1	98.3
L6-MP (mm)	29.36	26.2	31.9	28.2	­N/A	­N/A	40.2	38.2	­N/A	­N/A	­N/A	­N/A	37	33
U1-L1 (°)	129.77	129.81	121.9	122.5	­N/A	­N/A	­N/A	­N/A	­N/A	­N/A	­N/A	­N/A	117.7	117.4
Nasolabial angle	109.03	109.15	94.6	101.8	104.9	103.6	93.4	99	90.7	92.2	91.1	92	­N/A	­N/A
UL thickness at A point	16.7	14.35	15.4	13.3	­N/A	­N/A	16.5	13.9	­N/A	­N/A	­N/A	­N/A	­N/A	­N/A
UL thickness at VB (mm)	13.37	11.2	12.1	9.9	­N/A	­N/A	14.3	11.3	­N/A	­N/A	­N/A	­N/A	­N/A	­N/A
LL thickness at B point (mm)	11.87	10.67	13.5	11.3	­N/A	­N/A	14.8	12.4	­N/A	­N/A	­N/A	­N/A	­N/A	­N/A
UL to E-plane	-4.87	-5.12	­N/A	­N/A	-0.3	-1.5	­N/A	­N/A	-2.9	-2.5	-0.5	0	­N/A	­N/A
LL to E-plane	-2.29	-2.79	­N/A	­N/A	1.7	0.9	­N/A	­N/A	-0.3	0.9	1	1.4	­N/A	­N/A
UL protrusion	3.11	2.63	5.5	4.3	­N/A	­N/A	6.3	6.5	­N/A	­N/A	­N/A	­N/A	­N/A	­N/A
LL protrusion	2.7	1.9	5.3	4.6	­N/A	­N/A	5.6	6.1	­N/A	­N/A	­N/A	­N/A	­N/A	­N/A
Z-angle	77.47	78.69	73.9	74.7	­N/A	­N/A	69.5	67.5	­N/A	­N/A	­N/A	­N/A	N/A	N/A

In terms of gender comparison, the absence of a difference between males and females with respect to the angles SNA, SNB, and ANB illustrated by our sample of Lebanese adults is an uncommon finding in previous literature. Previous assessment of the Lebanese sample have noted a statistically significant difference in the SNB angle [28,29], which was smaller in females, and the ANB angle, which was larger in females [28]. Similar findings were noted in the Yemeni, Turkish, Saudi Arabian, and Sudanese populations [15,19,27] and also in assessments of Caucasians of North European ancestry [31]. Similar findings of a smaller SNB angle in females have been reported by Gu et al. [31] in the Chinese population and by Moldez et al. in the Filipino population [36], but in both of these assessments, ANB was nonetheless similar between males and females. Our findings are, however, similar to those of Al Zain and Ferguson [24] and Al-Awwad et al. [33], who found no statistically significant differences in any of the angles SNA, SNB, and ANB between males and females. On the other hand, our finding of smaller linear skeletal dimensions in the sagittal and vertical planes in Lebanese females (Co-A, Co-Gn, N-ANS, ANS-Me) corroborates previous research on Lebanese, Arab, Caucasian, and Asian populations [15,27-29,31,36].

The similarity in the dental components between Lebanese males and females, especially with respect to maxillary and mandibular incisor inclination, confirms the results reported by Ayoub et al. on a previous Lebanese sample [29]. Conversely, Uysal et al. [15] and Younso and Abuaffan [27] report more retroclined maxillary incisors and more proclined mandibular incisors in female Turks and Sudanese than in males, and more retroclined maxillary incisors and more retruded maxillary and mandibular incisors in Saudi Arabian females than in Saudi Arabian males. Mandibular incisors were similarly found more proclined in male Kuwaitis than in females [33], but the opposite finding was reported for the Yemeni population [19]. Finally, the similarity in upper and lower lip positions relative to E-plane echoes the findings of Uysal et al. [15] on the Turkish population and Younso and Abuaffan [27] on the Sudanese population, whereas other assessments in the region indicated a relatively more protrusive lower lip, on average, in Kuwaiti females than in males and a relatively more retrusive lower lip, on average, in Saudi Arabian females compared to males [15,33].

When considering the study’s limitations, the diversity in sampling techniques and in cephalometric analyses utilized in the published literature is one limitation for accurate comparisons between the different studies on various populations. Studies often differ in the age range, gender distribution, sample sizes, and variables analyzed, which can significantly influence the craniofacial measurements and lead to inconsistencies in the assessment of hard and soft tissues. In addition, the scarcity of comparable studies on multiple large samples on Lebanese adults particularly limits the adoption of new population-specific cephalometric norms. These methodological discrepancies increase the challenges when comparing craniofacial characteristics across different ethnicities.

To enable a more accurate description of cephalometric norms in various populations and in both genders, large collaborative studies across various populations are necessary, with a need to devise more objective criteria for the selection of normal representative samples of the populations studied, particularly limiting the subjectivity of recruiting participants who have “aesthetically pleasing profiles,” by developing quantifiable and reproducible selection guidelines. The concept of an esthetic profile is likely to differ not only between populations but also between researchers who may also have different beauty standards for males and females, a factor that affects the validity of any differences in cephalometric norms described between genders.

Finally, the increasing development and utilization of cone beam computed tomography and reconstructed cephalometric images similarly invites future assessments of hard and soft tissue cephalometric norms as measured on these reconstructed images.

## Conclusions

The clinical relevance of findings on craniofacial norms studies lies in how these studies provide a standardized reference for the evaluation of the craniofacial characteristics of a specific population. These findings help clinicians at different stages such as identification of individuals whose features significantly deviate from the norms related to the population they belong to, diagnosis of craniofacial syndromes and growth disturbances (in young patients), and treatment planning based on the amount of deviation from the norms related to a specific population, as well as limitations to achieving optimal treatment goals (for instance, surgical vs. non-surgical).

Prognosis of the treatment is highly influenced by the amount of deviation from the norms in terms of response to treatment. In fact, outcome prediction can improve the communication with the patients (predictable/non-predictable responses, relapse risk, future growth estimation, etc.). Craniofacial norms are actually clinical tools that improve decision-making for young and adult patients. When related to facial esthetics, they should be ethnicity-specific and gender-specific for optimal treatment outcomes and realistic goals. The findings in our study ascertain similarities of the Lebanese craniofacial features with Caucasians except in the mentioned variables, which is essential to provide references (that were still incomplete till date) for the concerned population.

Despite its limitations, the present work represents an important step towards the comprehensive description of hard and soft tissue cephalometric norms for the Lebanese population. The findings demonstrate that Lebanese adults with pleasant profiles and normal occlusion share many cephalometric similarities with Caucasian populations. However, Lebanese individuals tend to have smaller and more retrognathic jaws and a more obtuse nasolabial angle. No significant gender differences were observed in angular measurements related to the sagittal and vertical planes or in angular dental variables. On the other hand, linear skeletal and soft tissue measurements revealed significant gender-based differences, with Lebanese males displaying larger anterior skeletal base lengths, maxillary lengths, mandibular bodies, and greater nasofrontal and Z-angles.

Clinically, these population-specific craniofacial norms serve as essential diagnostic and planning tools. They allow orthodontists to identify deviations from expected norms, assess growth disturbances or craniofacial anomalies, and tailor treatment strategies (whether orthodontic or surgical) according to the degree of deviation. Moreover, such norms improve treatment predictability, facilitate prognosis estimation, and enhance communication with patients regarding outcomes and relapse risk. Given the role of facial esthetics in treatment planning, the establishment of ethnicity- and gender-specific standards is crucial. This study provides previously lacking normative data for the Lebanese population, supporting more individualized, evidence-based, and culturally appropriate orthodontic care.
